# Human gingival fibroblast secretome accelerates wound healing through anti-inflammatory and pro-angiogenic mechanisms

**DOI:** 10.1038/s41536-020-00109-9

**Published:** 2020-12-10

**Authors:** Parinaz Ahangar, Stuart J. Mills, Louise E. Smith, Stan Gronthos, Allison J. Cowin

**Affiliations:** 1 Future Industries Institute, University of South Australia, Adelaide, SA 5000 Australia; 2 Cell Therapy Manufacturing Cooperative Research Centre, Adelaide, SA 5000 Australia; 3 Adelaide Medical School, Faculty of Health and Medical Sciences, University of Adelaide, Adelaide, SA 5000 Australia; 4 South Australian Health and Medical Research Institute, Adelaide, SA 5000 Australia

**Keywords:** Cell biology, Stem cells

## Abstract

Healing of the skin and oral mucosa utilises similar mechanisms of tissue repair, however, scarring and the rate of wound closure is vastly superior in the oral cavity suggesting differences between these two environments. One key difference is the phenotype of dermal fibroblasts compared to fibroblasts of gingival tissues. Human gingival fibroblasts (hGFs) are undifferentiated cells with multi-differentiation and self-renewal capacities. This study aimed to examine if delivering hGFs or their secretome, contained in hGF-conditioned media (hGF-CM), would improve healing of the skin and recapitulate features of oral healing. Human fibroblasts, keratinocytes and endothelial cells were first treated with hGF-CM and showed improved migration, proliferation and angiogenic functions. A significant reduction in macroscopic wound area and histologic dermal wound width, as well as an increased rate of re-epithelialisation, were observed in both hGFs and hGF-CM treated murine excisional wounds. This improvement was associated with reduced inflammation, increased angiogenesis and elevated collagen deposition. These findings demonstrate that treatment of dermal wounds with either hGFs or hGF-CM may provide beneficial gingival-like properties to dermal wounds and may be a potential opportunity for improving healing of the skin.

## Introduction

In comparison with cutaneous wound repair, oral wounds have a more embryonic-like, scar-free healing response with decreased inflammation, faster re-epithelialisation and accelerated healing^1^. Generally, the healing of oral wounds proceeds through the same phases as cutaneous wounds, however, it is different in some other aspects such as moist external environment, presence of saliva, and different cell phenotypes^2^. Several studies have already shown that different fibroblast lineages contribute to skin and oral wound healing^3^. Gingival fibroblasts are more efficient at remodelling connective tissue than dermal fibroblasts^4^. Hence, it has been suggested that gingival fibroblasts may play an important role in the unique healing of oral wounds^5^.

Human gingival fibroblasts (hGFs) are the main cellular constituent of gingival tissue and are embryo-like cells with the capacity of self-renewal and clonogenicity^6^. Previous studies have reported that a minor proportion of hGFs are capable of multilineage differentiation including osteoblastic, chondrogenic, and adipogenic cellular differentiation in vitro and in vivo^6,7^. Cultured hGFs express mesenchymal stem cell (MSC)-associated cell surface markers including CD73, CD90, CD105, CD44 and STRO-1, but are negative for hemopoietic markers such as CD34 and CD45^8^. Importantly, hGFs also possess non-contact-dependent immunomodulatory properties associated with the secretion of anti-inflammatory cytokines^6,7^. The stem cell-like properties of GFs are mainly due to their subsets of pluripotent cells including gingiva-derived mesenchymal stem cells (GMSCs) and gingival multipotent progenitor cells (GMPCs)^9,10^. These characteristics of gingival fibroblasts have led to these stem cell-like cells being used for tissue repair and regeneration^11^.

In the past two decades, cellular therapies and particularly MSC therapies, have been widely promoted as potential skin wound treatments^12^. However, in order to generate therapeutically relevant doses, stem cells need to undergo extensive population doublings in vitro leading to an increase in the possibility of mutagenesis, cellular senescence, significant manufacturing costs and a decrease in cell function. Alternative cells, such as gingival fibroblasts, which are easily accessible, may serve as an attractive alternative to stem cells due to their high proliferative potential.

Although a number of studies have suggested a vital role for direct contact by stem cells for mediating their effects^13^, other studies suggest that only secreted factors are required for the immunomodulatory and regenerative effects of stem cells^14^. More recently, stem cell secretomes, generally in the form of conditioned media (CM), have been investigated as an alternative cell-free therapy in regenerative medicine^15,16^.

This paper aimed to investigate the effectiveness of treating wounds with hGFs and to subsequently assess its paracrine effect using hGF-conditioned media (hGF-CM). In vitro and in vivo investigations of skin cell proliferation, migration and functionality were determined as well as the cell and cell-free effects of hGFs on healing using a murine model of excisional wound repair.

## Results

### Treatment with hGFs and hGF-CM improve wound healing and enhance wound closure and re-epithelialisation in vitro and in vivo

**Fig. 1:**
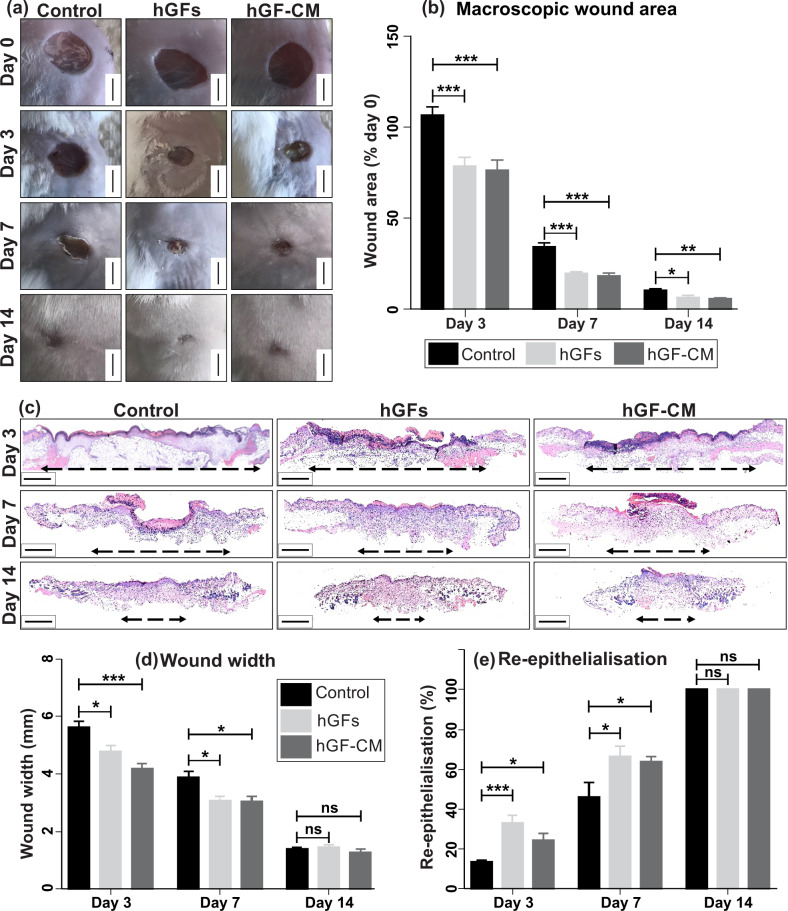
The effect of hGFs and hGF-CM on excisional wound healing. **a** Representative images of hGF, hGF-CM treated wounds at days 3, 7 and 14 of healing. Scale bars represent 5 mm. **b** Macroscopic area of excisional wounds at days 3, 7 and 14. *n* = 8. **c** Representative images of H&E stained wounds at days 3, 7 and 14 of healing. Scale bars represent 1 mm. **d** Histological wound width and **e** average re-epithelialisation of excisional wounds at days 3, 7 and 14. Images captured at ×10 objective. Black dashed lines indicate wound width. *n* = 8. **f** All data is represented as mean ± SEM. *p < 0.05, **p < 0.01, ***p < 0.001, ns = nonsignificant.

Excisional wounds intradermally injected with either 2 × 10^4^ hGFs or 20× concentrated hGF-CM showed significant improvements in macroscopic wound area on days 3, 7 and day 14 post-wounding in comparison to control-treated mice (Fig. 1a, b, Table 1). Histological evaluation of H&E stained wound sections (Fig. 1c) confirmed a decrease in wound width for both hGF and hGF-CM treatments compared to controls at days 3 and 7 of healing (Fig. 1d). The percentage of re-epithelialisation was also higher in hGF and hGF-CM treated wounds compared to controls at days 3 and 7 post-wounding and all wounds were fully re-epithelialized 14 days after wound induction (Fig. 1e, Table 1). Staining of HGF-injected wounds for the presence of anti-human nucleoli antibody (HNA) revealed positive human cells in the wound beds of the mice at days 3 and 7 confirming the delivery and retention of hGFs in the wounds (Supplementary Fig. 1).

**Table 1:** Assessment of excisional wound healing in hGFs and hGF-CM treated mice.

Healing assessment	Groups	Day 3	Day 7	Day 14
Macroscopic wound area (% initial wound)	Control	106.57 ± 4.58	34.23 ± 2.20	10.36 ± 0.82
	hGFs	78.53 ± 4.92	19.58 ± 1.01	6.31 ± 1.29
	*P* value	0.0010	<0.0001	0.0195
	hGF-CM	76.37 ± 5.55	18.15 ± 1.59	5.66 ± 0.57
	*P* value	0.0009	<0.0001	0.004
Microscopic wound width (mm)	Control	5.63 ± 0.20	3.87 ± 0.23	1.39 ± 0.06
	hGFs	4.79 ± 0.21	3.06 ± 0.15	1.45 ± 0.08
	*P* value	0.0125	0.0117	0.5633
	hGF-CM	4.18 ± 0.16	3.04 ± 0.17	1.26 ± 0.11
	*P* value	<0.0001	0.0133	0.3629
Re-epithelialisation (%)	Control	13.37 ± 1.2	46 ± 7.39	100 ± 0.0
	hGFs	33.18 ± 3.92	66.31 ± 5.12	100 ± 0.0
	*P* value	0.0003	0.0245	ns
	hGF-CM	21.37 ± 3.55	63.93 ± 2.43	100 ± 0.0
	*P* value	0.0434	0.0371	ns

**Fig. 2:**
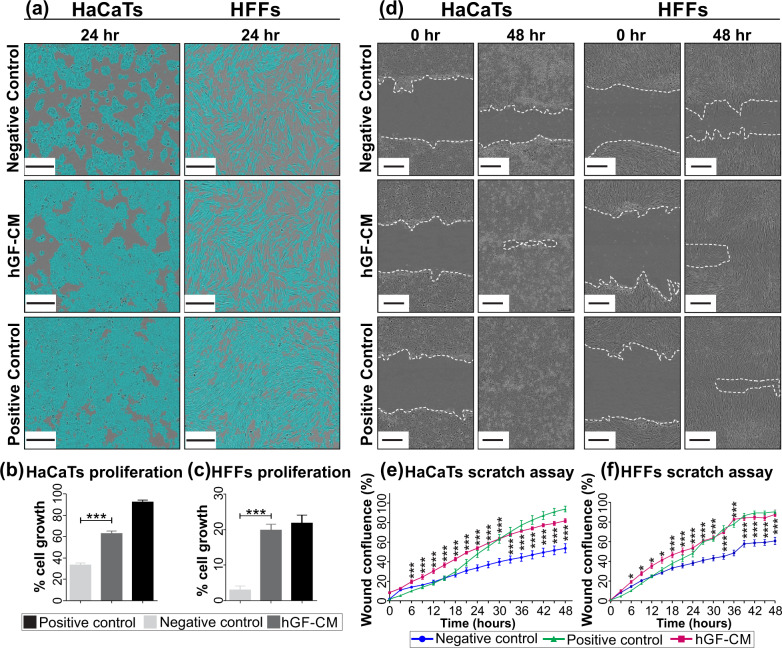
The effect of hGF-CM on fibroblast and keratinocytes. **a** Representative images of HaCaTs and HFFs 24 h after treatment with hGF-CM versus positive and negative control media. Scale bars represent 0.3 mm. **b**, **c** The effect of hGF-CM on the proliferation of (**b**) HaCaTs and (**c**) HFFs was quantified by Incucyte proliferation assay 24 h post seeding. *n* = 6. **d** Representative images of scratch assays show scratches immediately after the scratches had been made and then after 48 h. Scale bars represent 0.3 mm. **e**, **f** The effect of hGF-CM on the migration of (**e**) HaCaTs and (**f**) HFFs was measured using a scratch assay. *n* = 6. All data is represented as mean ± SEM. *p < 0.05, **p < 0.01, ***p < 0.001, ns = nonsignificant.

The paracrine effect of hGFs on cell proliferation and migration was determined in vitro using human keratinocytes (HaCaTs) and foreskin fibroblasts (HFFs). The addition of hGF-CM significantly increased proliferation of both HaCaTs by 30% (*p* < 0.0001) and HFFs by 16% (*p* < 0.0001) after 24 h of treatment with hGF-CM compared to the negative control (Fig. 2a–c). Similarly, HaCaTs and HFFs demonstrated significantly elevated migratory ability following 6 h of treatment with hGF-CM compared to the negative controls (Fig. 2d–f). Following 48 h after scratch, the wound confluence percentage of HFFs was 81.7% in hGF-CM treated cells while the wound closure of cells in negative control medium was 53.6% (*p* < 0.0001). Likewise, the confluence of HFFs in the presence of hGF-CM (87.8%) was higher than in negative control medium (60.7%, *p* < 0.0001).

### Treatment with hGFs and hGF-CM decrease the inflammatory response

**Fig. 3:**
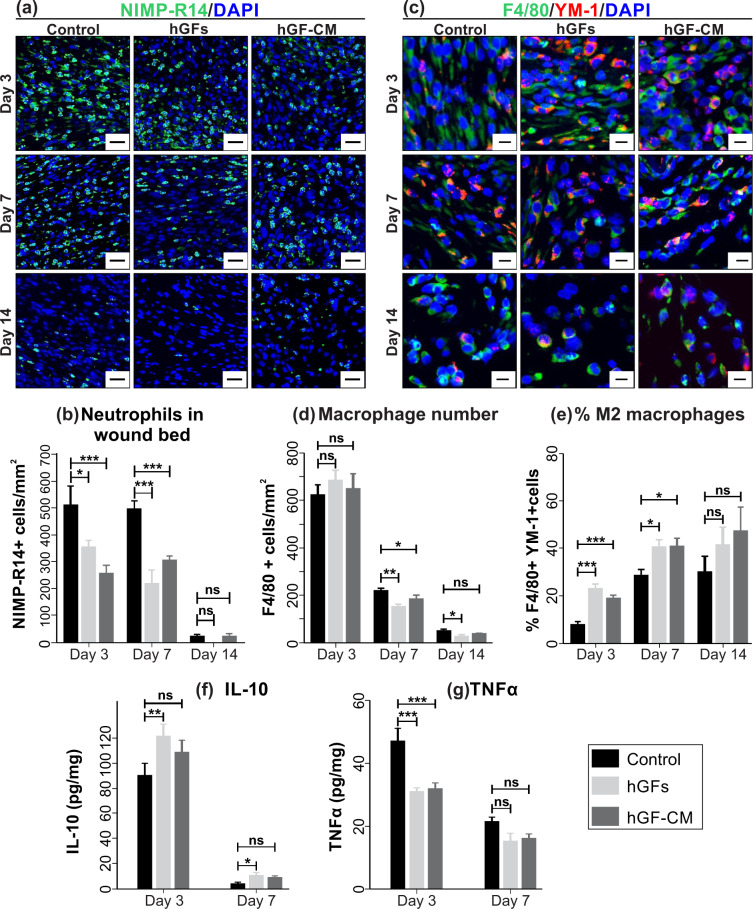
The effect of hGFs and hGF-CM on inflammation. **a**, **b** Immunofluorescent detection and quantification of NIMP + neutrophils in hGF and hGF-CM treated wounds. Scale bars represent 0.2 mm. **c**, **d** Immunofluorescent detection and quantification of F4/80+ macrophages in hGFs and hGF-CM treated wounds. **e** Quantification of the ratio of F4/80+ and YM-1 + M2 macrophages in hGF and hGF-CM treated and control wounds. Images captured at ×20 objective. Scale bars represent 0.1 mm. Quantification of **f** IL-10 level (pg of IL-10 in 1 mg of total protein in wounds) and **g** TNFα level (pg of TNFα in 1 mg of total protein in wounds) in the wound tissues using ELISA. *n* = 8. All data is represented as mean ± SEM. *p < 0.05, **p < 0.01, ***p < 0.001, ns = nonsignificant.

Immunofluorescent staining of wound tissues with the NIMP-R14 revealed a significant reduction in neutrophilic influx into the hGF treated wound sites on day 3 by 68.9% (*p* < 0.05) and on day 7 by 43.8% (*p* < 0.0001) compared to control wounds (Fig. 3a, b). Similarly, GF-CM treated wounds exhibited a reduction in neutrophil number on day 3 by 49.2% (*p* < 0.0001) and on day 7 by 61.1% (*p* < 0.0001) compared to control wounds (Fig. 3a, b). Treatment with hGF and hGF-CM were equally as effective at reducing inflammation and both were significantly lower than the respective controls. Note that the number of neutrophils in all wounds at day 14 was negligible across all treatments (Fig. 3a, b). Similarly, the quantification of macrophage infiltration using macrophage marker (F4/80) demonstrated a decrease in the presence of F4/80 positive macrophages in both hGF (*p* < 0.01) and hGF-CM (*p* < 0.05) treated wounds at day 7. This decrease in macrophage number was also observed at day 14 in hGF treated wounds (*p* < 0.05) but not wounds treated with hGF-CM (Fig. 3c, d). The percentage of macrophages expressing Ym-1, which is indicative of anti-inflammatory M2 macrophages, suggested that the rate of polarisation of macrophages to an anti-inflammatory state was increased following treatment with hGFs and hGF-CM at day 3 (*p* < 0.0001) and day 7 (*p* < 0.05) of healing (Fig. 3c, e).

To determine if the reduction in inflammatory cell numbers and increase in macrophage polarisation after treatment with hGFs and hGF-CM also altered cytokine production, the levels of interleukin 10 (IL-10) and tumour necrosis factor alpha (TNFα) were assessed by ELISA. The concentration of anti-inflammatory IL-10 in wounds treated with both hGFs and hGF-CM peaked compared to the control wounds. However, this upregulation was only statistically significant in hGF treated wounds at day 3 (*p* < 0.01) and 7 (*p* < 0.05) of healing (Fig. 3f). The level of pro-inflammatory TNFα was reduced after treatment with both hGFs and hGF-CM at both day 3 and day 7. This was highly significant at day 3 (*p* < 0.0001) compared to the control wounds indicating that both treatments were inhibiting the production of TNFα and flattening the inflammation phase (Fig. 3g).

### Treatment with hGFs and hGF-CM increase angiogenesis

**Fig. 4:**
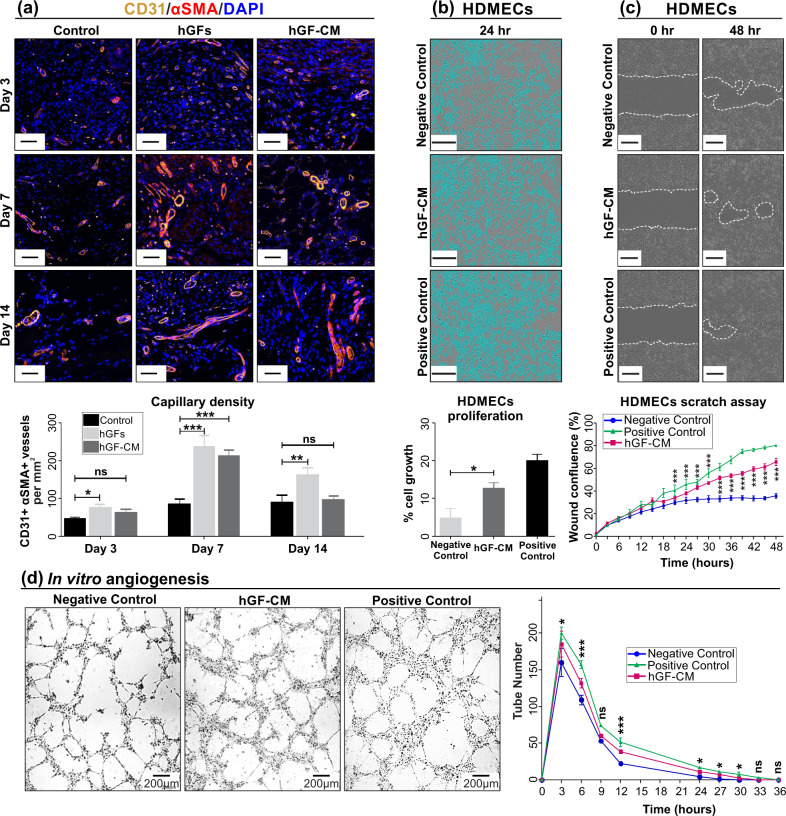
The effect of hGFs and hGF-CM on angiogenesis. **a** Immunofluorescent detection and quantification of CD31+ and αSMA+ mature blood vessels (structures) in hGFs and hGF-CM treated and control wounds. Images captured at ×20 objective. Scale bars represent 0.2 mm. *n* = 8. **b**–**d** The effect of hGF-CM treatment on HDMECs (**b**) proliferation, (**c**) migration and (**d**) angiogenic potency in vitro. Scale bars represent 0.3 mm. *n* = 6. All data are represented as mean ± SEM. *p < 0.05, **p < 0.01, ***p < 0.001, ns = nonsignificant.

To evaluate the effect of hGFs and hGF-CM on the wound vascularisation, tissues were stained with endothelial cell marker (CD31) and alpha-smooth muscle actin marker (αSMA). This dual staining localizes and quantifies mature blood vessels (CD31 + αSMA+) within the wound bed (Fig. 4a). A significant increase in capillary density was observed, as measured by the number of mature vessels per mm^2^, in hGF treated wounds compared to control wounds at day 3 (*p* < 0.05). Similarly, a significant increase in capillary density was observed at day 7 in both hGF and hGF-CM treated groups (*p* < 0.0001). There was also a higher number of blood vessels in hGF-treated wounds at day 14 of healing (*p* < 0.01, Fig. 4a). Treatment with hGF-CM encouraged HDMECs to proliferate, migrate and form significantly more vessel-like structures in vitro (Fig. 4b–d).

### Treatment with hGFs and hGF-CM augment the expression of collagen I and III

**Fig. 5:**
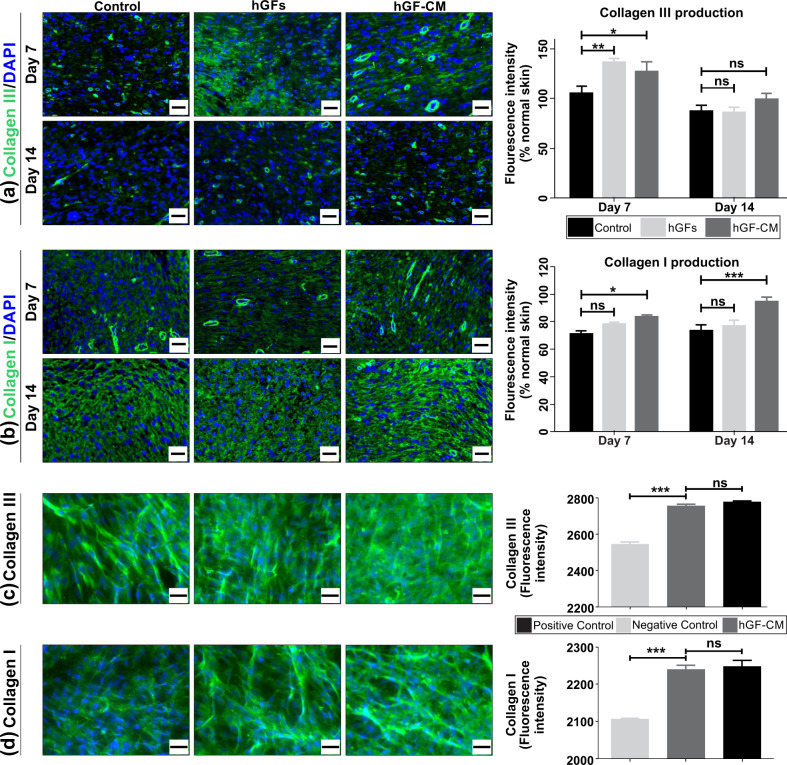
The effect of hGFs and hGF-CM on collagen deposition. **a**, **b** Immunofluorescent detection and quantification of (**a**) collagen III or (**b**) collagen I (green) counterstained with DAPI in hGF and hGF-CM treated and control wounds. Images captured at ×20 objective. Scale bars represent 0.2 mm. *n* = 8. **c**, **d** Immunofluorescent detection and quantification of (**c**) collagen III or (**d**) collagen I (green) expression in HFFs treated with hGF-CM and control media. Fluorescence intensities measured using CellSens dimension software. Images captured at ×20 objective. Scale bars represent 0.5 mm. *n* = 6. All data are represented as mean ± SEM. *p < 0.05, **p < 0.01, ***p < 0.001, ns = nonsignificant.

In order to assess the effect of hGF and hGF-CM treatments on the production of collagens, wound tissues were immunostained with both collagen I and collagen III antibodies (Fig. 5a, b). As indicated in Fig. 5a, collagen III expression was increased in response to hGFs (*p* < 0.01) and hGF-CM (*p* < 0.05) on day 7 compared to controls. However, by day 14, all treatments resulted in a similar level of Collagen III expression. Elevated expression of collagen I was observed in hGF-CM treated wounds at day 7 (*p* < 0.05) and 14 (*p* < 0.0001) days after wound induction. Interestingly, there was no significant difference in the amount of Collagen I in hGF treated wounds compared to the control wounds (Fig. 5b). In vitro, hGF-CM treated HFFs demonstrated significantly augmented expression of collagen III (*p* < 0.0001, Fig. 5c) and collagen I (*p* < 0.0001, Fig. 5d) compared to the negative control. Myofibroblasts deposit extracellular collagen and contribute to wound contraction. Persistence of myofibroblasts in wounds can contribute to fibrosis and scar formation^17^. Assessment of myofibroblast marker (αSMA) revealed no significant differences in treated and control wounds at either day 7 or day 14 post-wounding indicating that none of these treatments specifically induce differentiation of fibroblasts into pro-scarring, contractile myofibroblasts (Supplementary Fig. 2).

### Analysis of hGF secretome

Considering the beneficial effects of hGF-CM on skin cell functions and wound healing, bead array analysis was used to identify proteins present within the CM, which may be contributing to these effects. Secretome analysis revealed high levels of inflammation-related cytokines including IL-6, Arginase, MCP-1 and IL-8. Furthermore, the presence of growth factors and ECM proteins such as HGF, FGF-2, VEGF, Ang-1, Ang-2, MMP-2, MMP-9 and TIMP-1 was detected as were adhesion molecules (VCAM-1, CD166 and CD44) (Table 2).

**Table 2:** hGF protein secretome.

Protein ID	Concentration (pg/ml)
Matrix metalloproteinases	(MMP-2)/12464 ± 730, (MMP-9)/455 ± 46
Tissue inhibitor of metalloproteinase (TIMP-1)	>771464
Hepatocyte growth factor (HGF)	362 ± 38
Transforming growth factor-β1 (TGF-β1)	9 ± 2
Vascular endothelial growth factor (VEGF)	59 ± 6
Platelet-derived growth factor-AA (PDGF-AA)	15 ± 1
Fibroblast growth factor-2 (FGF-2)	270 ± 79
Angiopoietin-1 (Ang-1)	4616 ± 303
Angiopoietin-2 (Ang-2)	480 ± 50
Vascular cell adhesion protein (VCAM-1)	535 ± 60
CD166	5460 ± 851
CD44	9015 ± 716
Monocyte chemoattractant protein-1 (MCP-1)	3498 ± 120
Interleukins	IL-6/15038 ± 1289, IL-8/345 ± 130, IL-2/25 ± 4, IL-1β/12 ± 2, IL12p40/ 141.51, IL23/ 62.74
Arginase	19862 ± 4771

Functionally distinct classes of molecules identified by bead array assays.

## Discussion

It is well known that hGFs have stem cell-like properties and play a key role in promoting healing of the oral mucosa^18^. In this study, hGFs and hGF-CM enhanced cutaneous wound healing, as evidenced by faster re-epithelialization, decreased inflammation, increased angiogenesis and elevated collagen deposition. The decrease in wound area and width was similar to reports on the effect of MSCs and their conditioned media on the wound width of acute wounds in mice^19^. The addition of hGF-CM promoted migration, proliferation and collagen deposition of dermal fibroblasts in culture suggesting that hGFs and hGF-CM may affect wound healing via the promotion of fibroblast behaviour in the wound area. Accelerated re-epithelialisation in response to hGFs and hGF-CM was supported by in vitro effects of hGF-CM on the regulation of epithelial restoration through increasing the rate of proliferation and migration of keratinocytes. The effect of hGFs on skin cells may be explained by their ability to secrete proteins associated with cell proliferation and migration including TGF-β1, FGF-2 and hepatocyte growth factor^20^.

Previous studies have shown the suppressing effect of gingiva-derived stem cells on T cells, mast cells, dendritic cells and macrophages^21–23^. Similarly, in the present study, wounds injected with hGF and hGF-CM showed a dampened inflammatory response as evidenced by significantly lower expression of the neutrophils and macrophages in the wound bed. Macrophages initially have a pro-inflammatory phenotype (M1 macrophages) that enables the killing of pathogens. The macrophages then differentiate to become anti-inflammatory M2 macrophages, which operate in constructive processes like cell proliferation and tissue repair^24^. M1 macrophages are required at the early stages of healing and inflammation while M2 macrophages play an important role in granulation tissue formation and angiogenesis. Macrophage polarisation (M1 to M2) has been shown to improve wound healing and regeneration^25^. In this study, treatment with both hGFs and hGF-CM promoted polarisation of the macrophages towards the M2 phenotype. This finding is consistent with a study on human gingiva‐derived mesenchymal stem cells in which these cells enhanced wound healing by accelerating the polarisation of M2 macrophages^26^. The pro-inflammatory cytokine TNFα was also decreased in hGF and hGF-CM treated wounds. High levels of TNFα during wound repair lead to a prolonged inflammatory response and can contribute to impaired healing responses^27^. Therefore, the reduced expression of TNFα observed in hGF and hGF-CM treated wounds could contribute to the improved healing that was observed in this study. In contrast, IL-10 was found to be elevated in hGF and hGF-CM treated wounds. IL-10 is an anti-inflammatory and antifibrotic cytokine that modulates neutrophils and monocyte infiltration as well as helps to promote M2 macrophage polarisation^28,29^. Increased IL-10 in the treated wounds could therefore help to promote a conducive environment for successful wound repair. A number of inflammation-related proteins such as arginase, MCP-1, IL-6, IL-8, IL-12p40, IL-23 and TARC were also found to be present in the secretome of hGFs. Given this, the direct effect of hGFs and the indirect effect of the hGF-CM could have significant effects on dampening of the inflammatory response which is how they have an overall positive effect on wound repair.

The number of mature blood vessels as determined by αSMA positive perivascular cells and CD31 positive endothelial cells was increased in both hGF and hGF-CM treated wounds indicating a potential direct effect on angiogenesis. This improvement was most obvious during the early stages of healing in response to hGF-CM but was for a longer duration in wounds treated with hGFs. Assessment of hGF-CM found several secreted proangiogenic factors including VEGF, Ang-1 and Ang-2 which can enhance vascularisation and angiogenesis in the wound bed^30^. Furthermore, hGF-CM increased proliferation, migration and the angiogenic response of human endothelial cells in vitro suggesting paracrine modulation of endothelial cells behaviour as the main angiogenic function of hGFs.

Collagen synthesis is an important process during wound healing; however, delayed or prolonged collagen deposition can contribute to the formation of chronic wounds and scars respectively. Therefore, the promotion of balanced collagen deposition would be desirable for a successful cell therapy^31^. In this study, collagen synthesis by human dermal fibroblasts was increased in the presence of hGF-CM in vitro. Both hGF and hGF-CM treated wounds also displayed higher expression levels of collagen I and collagen III. Moreover, hGF treatment increased collagen III production at early stages of wound healing but had little effect on collagen I, resulting in a higher ratio of collagen III/I which would be indicative of an improved and potentially scar-free healing response. No effect of hGF or hGF-CM was observed on the presence of myofibroblasts within the wounds suggesting there these treatments did not specifically induce differentiation of fibroblasts into pro-scarring, contractile myofibroblasts.

In conclusion, treatment of wounds with either cells or cell-free conditioned media enhanced healing with a significant decrease in dermal wound width and increased rate of re-epithelialisation. Treatment with cell-free conditioned media was equally as effective as the cell therapy with no significant difference being observed between the effects of these treatments using either macroscopic or histological assessments. Inflammation within the wound bed was decreased with both hGFs and hGF-CM while angiogenesis and collagen deposition were improved. Although both treatments had a positive impact on wound healing, our findings suggest that the cell-free approach could be effective to take forward for development as a wound treatment as it is potentially safer, easier to manufacture, store and deliver to wounds without the hazards and limitations of cell-based treatments.

## Methods

### Cell culture

Human Gingival Fibroblasts (hGFs) were obtained from the Mesenchymal Stem Cell Research Group (SAHMRI, Adelaide, Australia) and characterised as described previously^8^. hGFs were cultured in Alpha modified Eagle’s minimum essential medium (α-MEM) with 10% foetal bovine serum (FBS, Atlas biologicals, Fort Collins, USA), 2 mM L-glutamine (Lonza, Basel, Switzerland), 100 mM L-ascorbate-2-phosphate, 50 U/mL penicillin and 50 U/mL streptomycin (Lonza, Basel, Switzerland), and 1 mM sodium pyruvate (Thermo Fisher Scientific, VIC, Australia). The hGFs were seeded at a density of 5 × 10^3^ cells per cm^2^ on T75 culture flasks at 37 °C in a humidified incubator with 5% CO_2_. Human foreskin fibroblasts (HFFs; CellBank Australia, Westmead, Australia) and human immortalized keratinocytes (HaCaTs; AddexBio, San Diego, CA, USA) were cultured in Low-glucose DMEM medium (Thermo Fisher Scientific, VIC, Australia) with 2 mM L-Glutamine (Lonza, Basel, Switzerland), 10% FBS (Atlas Biologicals, Fort Collins, USA), and 100 U/mL penicillin/streptomycin (Lonza, Basel, Switzerland). In addition, human dermal microvascular endothelial cells (HDMECs PromoCell, Heidelberg, Germany) were cultured in endothelial cell growth medium MV2 (PromoCell) with 2 mM L-Glutamine (Lonza, Basel, Switzerland), 10% FBS (Atlas Biologicals, Fort Collins, USA), and 100 U/mL penicillin/streptomycin (Lonza, Basel, Switzerland). All cells were incubated at 5% CO_2_, 37 °C and 95% humidity.

### Conditioned medium collection

The hGFs were derived from adult human gingival tissues and characterised as previously described^8^. The hGFs were cultured as described in detail in the previous section and upon reaching 80% confluency, their medium was removed, and the cells washed 5 times with PBS to remove serum. 10 mL of serum-free DMEM medium was then added to each flask. Following 24 h incubation, the hGF-CM was collected, centrifuged and sterilized using 0.22 µm filters. The conditioned medium was concentrated 20× using GE Vivaspin™ Protein Concentrator Spin Columns (Bio-Strategy, Melbourne, Australia). In all experiments, fresh serum-supplemented DMEM medium (10% FBS) was used as the positive control while serum-free DMEM medium was used as the negative control.

### Proliferation

HFFs, HaCaTs and HDMEC (7 × 10^3^ cells/well) were seeded into 96-well plates, incubated at 37 °C and the cells were photographed after 24 h using a ×10 objective. A confluency mask was used to measure cell confluence in each well using IncuCyte ZOOM® Software (2015A, REV1, Essen Bioscience, Ann Arbor, MI, USA).

### Migration

Confluent cell monolayers were scratched using a WoundMaker™ (Essen Bioscience, Ann Arbor, MI, USA), washed twice using PBS and the media was replaced with either control media or hGF-CM. Images were taken immediately after scratching and every 3 h up to 48 h. The migration rate was obtained by measuring the percentage of cell confluence of each scratch area using IncuCyte ZOOM® Software as above.

### Angiogenesis

HDMECs (1.5 × 10^4^) suspended in 50 µL of either control media or hGF-CM were seeded in growth factor reduced Matrigel-coated wells (Corning Life Science, New York, USA). The number of the tubes in each well were counted every 3 h over 36 h or until all tubes were degraded.

### In vitro assessment of collagen I and III synthesis

Collagens I and III were determined using a 96-well immunohistochemistry assay^32^. HFFs were seeded in DMEM with 10% FBS at 5 × 10^4^ cells/well into 96-well plates. The medium was replaced with hGF-CM or control media after 24 h. Plates were left in the incubator for 60 h to allow collagens to be deposited. Cells were washed with PBS, fixed and permeabilised in methanol (−20 °C). They were then treated with Tween 20 (0.5%) in PBS for 10 min before incubating in 3% normal goat serum for 30 min and finally incubating with primary antibodies (rabbit anti-human collagen type I (5 µg/mL, Rockland Immunochemicals, Pennsylvania, USA), rabbit anti-human collagen type III (5 µg/mL, Rockland Immunochemicals, Pennsylvania, USA)) at room temperature for 2 h. Cells were incubated with secondary antibody Alexa Fluor 488 for an hour, stained for 2 min with 1:5000 DAPI, left in PBS and imaged using CellSens software (Olympus, Tokyo, Japan).

### Animal studies

These studies were conducted with approval from the University of South Australia Animal Ethics Committee following the Australian Code of Practice for the Care and the Use of Animals for Scientific Purposes (AEC: U15/19). The dorsum of 10–12-week-old female BALB/c mice (ARC, Perth, Australia) was shaved and cleansed before they received two 6 mm^2^ dorsal excisional wounds at 5 mm either side of the midline and 10 mm from the base of the skull. Power studies showed that a sample size of 8 would give 90% power using a 5% test level and a one-tailed test. Depending on grouping, 2 × 10^4^ hGFs suspended in 100 µl DMEM were delivered by intradermal injection around the margins of each wound. Alternatively, 100 µl of 20× concentrated hGF-CM was delivered to each wound margin, or 100 µl DMEM medium without FBS was injected intradermally to the control group. In each case, 25 µl was injected intradermally at 4 points around the wound margins. The mice were euthanized at 3, 7 or 14 days post-wounding. A ruler was aligned next to the wound to allow direct macroscopic measurement of wound area and digital photographs were taken. The wounds were surgically excised to the fascia and processed for histology and immunohistochemistry. Digital images of the wounds at each timepoint were analysed to determine macroscopic wound area.

### Histological evaluation and immunohistochemistry

Histological sections (4 µm) from paraffin-embedded fixed tissues were stained with haematoxylin-eosin (H&E) for microscopic analysis of wound healing. Moreover, sections were subjected to immunohistochemistry following antigen retrieval as described previously^33^. Tissue sections were blocked in 3% blocking serum for 30 min at room temperature, incubated with rabbit anti-human collagen type I (5 µg/mL, Rockland Immunochemicals, Pennsylvania, USA), rabbit anti-human collagen type III (5 µg/mL, Rockland Immunochemicals, Pennsylvania, USA), rat neutrophil marker (NIMP-R14, 0.5 µg/mL, Santa Cruz Biotechnology, Texas, USA) and rabbit anti-CD31 (0.2 µg/mL, Abcam, Cambridge, UK), F4/80 (5 µg/mL, Bio-Rad, NSW, Australia), YM-1 (0.37 µg/mL, StemCell Technologies, Vancouver, Canada), actin smooth muscle antibody (αSMA, 0.7 µg Agilent Technologies, VIC, Australia) antibodies overnight at 4 °C. Subsequently, tissue sections were stained with secondary goat anti-rabbit IgG, Alexa Fluor 488, goat anti-rabbit IgG, Alexa Fluor 568 or goat anti-rat IgG Alexa Fluor 488 (5 µg/mL, Invitrogen, CA, USA). To visualize nuclei and overall tissue architecture, sections were stained for 5 min in 4′,6-diamidino-2-phenylindole (DAPI, 1:5000 of 1 mg/mL stock). Sections were imaged with Olympus IX81 microscope (Olympus, Tokyo, Japan).

### Enzyme-linked immunosorbent assay (ELISA)

The wound tissues were homogenized in 1:20 T-per protein extraction reagent (Thermo Fisher Scientific, VIC, Australia) using MoBio BeadMill tubes (Ceramic Bead Tubes, MoBio, CA, USA) in the Bullet Blender Storm (Next Advance, NY, USA). Total amount of protein of each tissue was measured using NanoDrop Lite Spectrophotometer (Thermo Fisher Scientific, VIC, Australia). Tissue lysates were diluted 1:4 in assay buffer and the amount of mouse IL-10, mouse TNFα in wound tissues were quantified by enzyme-linked immunosorbent assay (ELISA, BioLegend, CA, USA) according to the manufacturer’s protocols (MAX™ Deluxe Set Mouse IL-10 # 431414, MAX™ Deluxe Set Mouse TNFα # 430904. Results were acquired by measuring absorbance at 450 nm using a plate reader (Tecan Group, Mannedorf, Switzerland).

### Bead array assay

The bead-based immunoassay was carried out according to the manufacturer’s protocols (BioLegend’s LEGENDplex™, San Diego, CA, USA). Concentrated hGF-CM and standards were incubated with protein-specific antibody-conjugated capture beads. Following binding of the target proteins with their capture beads, biotinylated detection antibodies were added to the wells and incubated for an hour. Streptavidin phycoerythrin was added to the wells and incubated for 30 min. After washing with SA-PE, the wells were loaded with wash buffer and read using a BD LSRFortessa™ flow cytometer (BD Bioscience, North Ryde, Australia).

### Statistical analysis

The results are presented as mean ± SEM. Data analysis was performed using GraphPad Prism (Graphpad, San Diego, CA, USA). One-way and two-way analysis of variance (ANOVA) tests were used for multiple comparisons followed by Tukey and Bonferroni post-tests. A value of *p* < 0.05 was set for the significance value.

### Reporting summary

Further information on experimental design is available in the Nature Research Reporting Summary linked to this paper.

## Supplementary information

Supplementary Information

Reporting Summary Checklist

## Data Availability

All data generated or analysed during this study are included in this published article and its Supplementary Information files.
