# Supplementary material for: Human gingival fibroblast secretome accelerates wound healing through anti-inflammatory and pro-angiogenic mechanisms
Source: NPJ Regen Med. 2020 Dec 10;5:24. doi: 10.1038/s41536-020-00109-9 (PMC7728777; doi:10.1038/s41536-020-00109-9)
Supplement: Supplementary file 1 — Supplementary Information [file 41536_2020_109_MOESM1_ESM.pdf]

Supplementary Information

Supplementary Note 1: Murine wounds injected with human gingival fibroblasts display the presence of human nucleolar antigen at day 3, 7 and 14.

In order to visualize the injected hGFs in each time-point, the paraffin-embedded wound tissues treated with 2×10^4 hGF cells in 100µl DMEM or just 100µl DMEM (control group) were stained with anti-human nucleoli antibody (HNA). hGF-injected wounds displayed the presence of HNA positive cells in the wound bed at day 3 and 7 of healing confirming the delivery and retention of hGFs in the wound. The number of positive cells in day 14 wounds was negligible (Supplementary Figure 1).

Supplementary Note 2: Treatment of wounds with hGF and hGF-CM has no impact on αSMA positive myofibroblasts.

To determine if hGF and hGF-CM treatments impact myofibroblast differentiation within the wounds, αSMA expression was examined in treated and control wounds. αSMA expression was quantified using immunohistochemistry staining of wound tissues with αSMA antibody. No significant differences were observed in the level of αSMA in hGF, hGF-CM-treated and control wounds at day 7 and 14 of healing (Supplementary Figure 2).

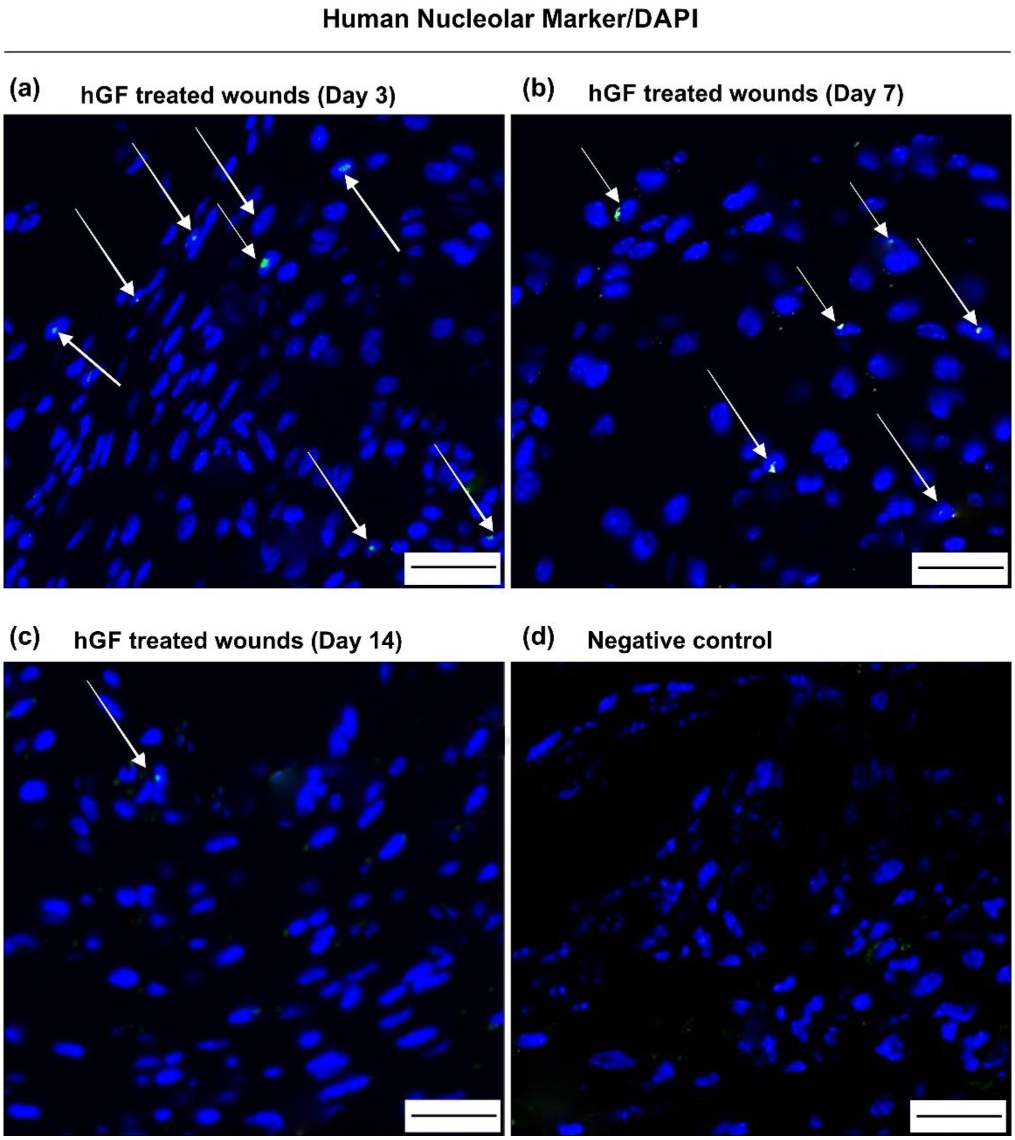

Supplementary Figure 1: Tracking human cells in hGF-injected excisional wounds. The immunofluorescent human cells by HNA (green) staining counterstained with DAPI (blue) in 2×10^4 hGF-injected wounds on (a) day 3, (b) 7 and (c) 14 of healing. (d) DMEM-injected control wounds. Images captured at 20X objective. Scale bars represent 0.25mm.

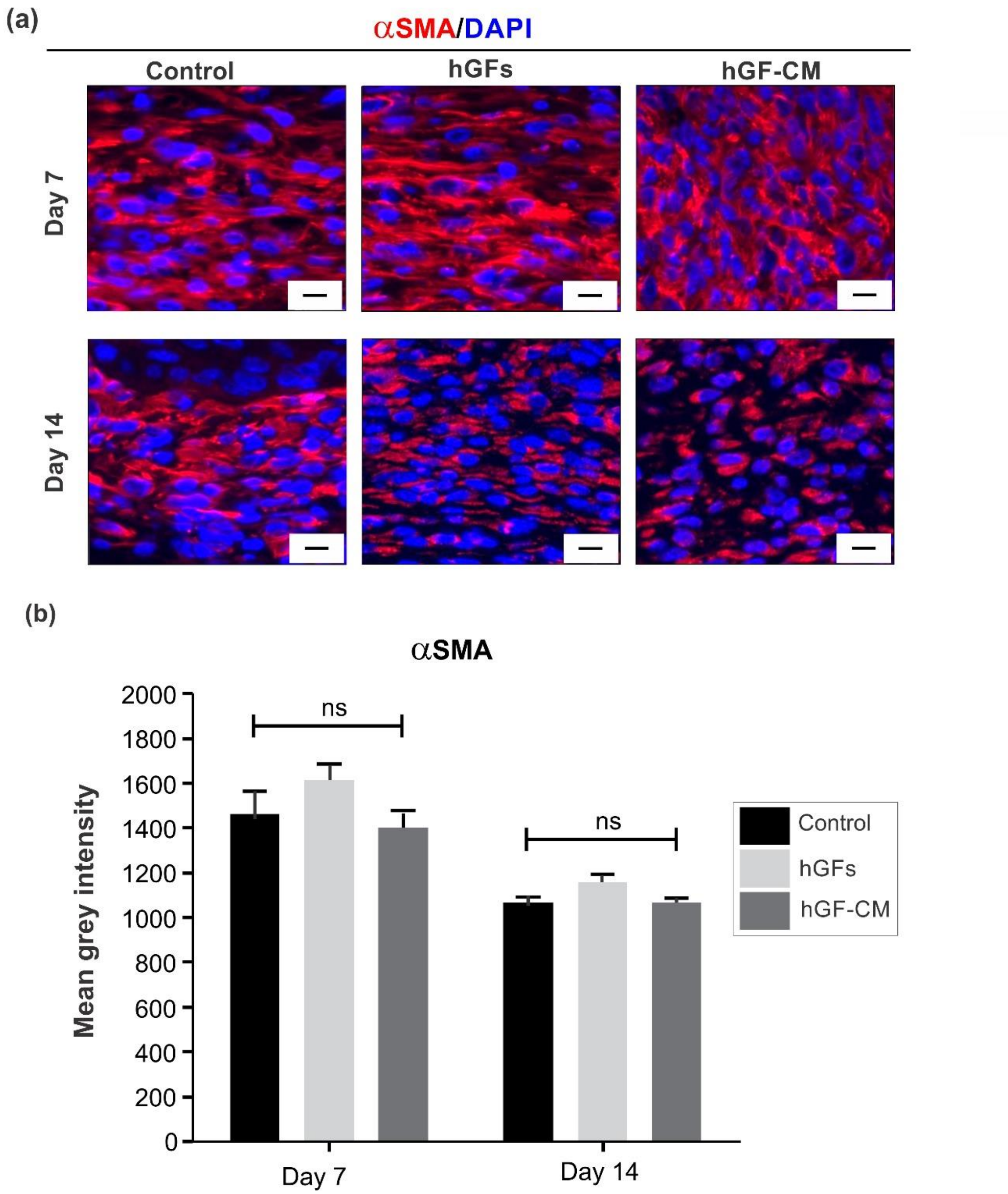

Supplementary Figure 2: The effect of hGFs and hGF-CM on αSMA positive myofibroblasts. a,b) Immunofluorescent detection and quantification of αSMA (red) counterstained with DAPI in hGF and hGF-CM treated and control wounds. Images captured at 20X objective. Scale bars represent 0.1mm. n= 8. Fluorescence intensities using CellSens dimension software. All data is represented as mean ± SEM. ns= non-significant.
