# Supplementary material for: Human gingival fibroblast secretome accelerates wound healing through anti-inflammatory and pro-angiogenic mechanisms
Source: NPJ Regen Med. 2020 Dec 10;5:24. doi: 10.1038/s41536-020-00109-9 (PMC7728777; doi:10.1038/s41536-020-00109-9)
Supplement: Supplementary file 2 — Reporting Summary Checklist [file 41536_2020_109_MOESM2_ESM.pdf]

nature research

Corresponding author(s): Allison Cowin
Last updated by author(s): 02/10/2020

Reporting Summary

Nature Research wishes to improve the reproducibility of the work that we publish. This form provides structure for consistency and transparency in reporting. For further information on Nature Research policies, see our Editorial Policies and the Editorial Policy Checklist.

Statistics

For all statistical analyses, confirm that the following items are present in the figure legend, table legend, main text, or Methods section.

n/a	Confirmed	
☐	☒	The exact sample size (n) for each experimental group/condition, given as a discrete number and unit of measurement
☒	☐	A statement on whether measurements were taken from distinct samples or whether the same sample was measured repeatedly
☐	☒	The statistical test(s) used AND whether they are one- or two-sided<br>*Only common tests should be described solely by name; describe more complex techniques in the Methods section.*
☒	☐	A description of all covariates tested
☒	☐	A description of any assumptions or corrections, such as tests of normality and adjustment for multiple comparisons
☐	☒	A full description of the statistical parameters including central tendency (e.g. means) or other basic estimates (e.g. regression coefficient) AND variation (e.g. standard deviation) or associated estimates of uncertainty (e.g. confidence intervals)
☒	☐	For null hypothesis testing, the test statistic (e.g. F, t, r) with confidence intervals, effect sizes, degrees of freedom and P value noted<br>*Give P values as exact values whenever suitable.*
☒	☐	For Bayesian analysis, information on the choice of priors and Markov chain Monte Carlo settings
☒	☐	For hierarchical and complex designs, identification of the appropriate level for tests and full reporting of outcomes
☒	☐	Estimates of effect sizes (e.g. Cohen's d, Pearson's r), indicating how they were calculated

Our web collection on statistics for biologists contains articles on many of the points above.

Software and code

Policy information about availability of computer code

Data collection: *Provide a description of all commercial, open source and custom code used to collect the data in this study, specifying the version used OR state that no software was used.*

Data analysis: *Provide a description of all commercial, open source and custom code used to analyse the data in this study, specifying the version used OR state that no software was used.*

For manuscripts utilizing custom algorithms or software that are central to the research but not yet described in published literature, software must be made available to editors and reviewers. We strongly encourage code deposition in a community repository (e.g. GitHub). See the Nature Research guidelines for submitting code & software for further information.

Data

Policy information about availability of data

All manuscripts must include a data availability statement. This statement should provide the following information, where applicable:

- Accession codes, unique identifiers, or web links for publicly available datasets
- A list of figures that have associated raw data
- A description of any restrictions on data availability

All data generated or analysed during this study are included in this published article and its Supplementary Information files.

Field-specific reporting

Please select the one below that is the best fit for your research. If you are not sure, read the appropriate sections before making your selection.

☒ Life sciences ☐ Behavioural & social sciences ☐ Ecological, evolutionary & environmental sciences

For a reference copy of the document with all sections, see nature.com/documents/nr-reporting-summary-flat.pdf

Life sciences study design

All studies must disclose on these points even when the disclosure is negative.

Sample size	All experiments were conducted with 90% power and a significance of 0.05.
Data exclusions	NA
Replication	In vitro data were obtained from three independent experiments.
Randomization	Mice were randomly allocated into treatment groups and control group.
Blinding	All in vivo studies and assessments were blinded.

Reporting for specific materials, systems and methods

We require information from authors about some types of materials, experimental systems and methods used in many studies. Here, indicate whether each material, system or method listed is relevant to your study. If you are not sure if a list item applies to your research, read the appropriate section before selecting a response.

Materials & experimental systems

n/a	Involved in the study	
☐	☒	Antibodies
☐	☒	Eukaryotic cell lines
☒	☐	Palaeontology and archaeology
☐	☒	Animals and other organisms
☒	☐	Human research participants
☒	☐	Clinical data
☒	☐	Dual use research of concern

Methods

n/a	Involved in the study	
☒	☐	ChIP-seq
☒	☐	Flow cytometry
☒	☐	MRI-based neuroimaging

Antibodies

Antibodies used	Rabbit anti-human collagen type I (5 µg/mL, Rockland Immunochemicals, Pennsylvania, USA), rabbit anti-human collagen type III (5 µg/mL, Rockland Immunochemicals, Pennsylvania, USA), rat neutrophil marker (NIMP-R14, 0.5 µg/mL, Santa Cruz Biotechnology, Texas, USA) and rabbit anti-CD31 (0.2 µg/mL, Abcam, Cambridge, UK), F4/80 (5µg/mL, Bio-Rad, NSW, Australia), YM-1 (0.37 µg/mL, StemCell technologies, Vancouver, Canada), actin smooth muscle antibody (αSMA, 0.7 µg Agilent Technologies, VIC, Australia), goat anti-rabbit IgG, Alexa Fluor 488, goat anti-rabbit IgG, Alexa Fluor 568 or goat anti-rat IgG Alexa Fluor 488 (5 µg/mL, Invitrogen, CA, USA).
Validation	Each antibody was validated according to manufacturers protocols.

Eukaryotic cell lines

Policy information about cell lines

Cell line source(s)	Human Foreskin Fibroblasts (HFFs) and human keratinocytes (HaCaTs) were obtained from ATCC, USA. Human dermal microvascular endothelial cells (HDMECs) were obtained from PromoCell, Heidelberg, Germany.
Authentication	Human Foreskin Fibroblasts (HFFs) and human keratinocytes (HaCaTs) were obtained from ATCC, USA. Human dermal microvascular endothelial cells (HDMECs) were obtained from PromoCell, Heidelberg, Germany.
Mycoplasma contamination	All cell lines were tested negative for mycoplasma contamination using LookOut® Mycoplasma PCR Detection Kit, Sigma-Aldrich, NSW, Australia.
Commonly misidentified lines (See ICLAC register)	*Name any commonly misidentified cell lines used in the study and provide a rationale for their use.*

Animals and other organisms

Policy information about studies involving animals; ARRIVE guidelines recommended for reporting animal research

Laboratory animals	10-12-week-old female BALB/c mice (ARC, Perth, Australia).
Wild animals	*Provide details on animals observed in or captured in the field; report species, sex and age where possible. Describe how animals were caught and transported and what happened to captive animals after the study (if killed, explain why and describe method; if released, say where and when) OR state that the study did not involve wild animals.*
Field-collected samples	*For laboratory work with field-collected samples, describe all relevant parameters such as housing, maintenance, temperature, photoperiod and end-of-experiment protocol OR state that the study did not involve samples collected from the field.*
Ethics oversight	These studies were conducted with approval from the University of South Australia Animal Ethics Committee following the Australian Code of Practice for the Care and the Use of Animals for Scientific Purposes (AEC: U15/19).

Note that full information on the approval of the study protocol must also be provided in the manuscript.
